# Trends and Factors Associated With Risk Perception, Anxiety, and Behavior From the Early Outbreak Period to the Controlled Period of COVID-19 Epidemic: Four Cross-Sectional Online Surveys in China in 2020

**DOI:** 10.3389/fpubh.2021.768867

**Published:** 2022-01-18

**Authors:** Bei Liu, Hanyu Liu, Bingfeng Han, Tianshuo Zhao, Tao Sun, Xiaodong Tan, Fuqiang Cui

**Affiliations:** ^1^Department of Laboratorial Science and Technology & Vaccine Research Center, School of Public Health, Peking University, Beijing, China; ^2^Department of Health Policy and Management, School of Medicine, Hangzhou Normal University, Hangzhou, China; ^3^Department of Occupational and Environmental Health, School of Public Health, Wuhan University, Wuhan, China

**Keywords:** COVID-19 pandemic, anxiety, perceived risk, protective behavior, cross-sectional study design

## Abstract

**Background:** The first wave of the COVID-19 epidemic in China was brought under with 3 months—from mid-January 2020 to the end of March 2020. Less studies examined dynamic psychological effect and behaviors during COVID-19 pandemic. This study aims to examine perceived risk, anxiety, and behavioral response of the general public related to the outbreak of COVID-19 in four cross-sectional surveys conducted throughout China.

**Methods:** In 2020, four cross-sectional, population-based online survey were conducted from January 28 to February 3, from February 10 to 12, from February 20 to 22, and from March 1 to 10, respectively. Convenience sampling was used for easy recruiting survey participants under the long-term impact of the COVID-19 epidemic. The four independent online questionnaires were sent from the same approach (WeChat and MicroBlog), and anyone who receives the questionnaire on the Internet or mobile phone and meets the inclusion criteria could fill in it. The same questionnaires repeatedly used in the four surveys. Socio-demographic information and individual protective practice were collected and the state-trait anxiety inventory (STAI) was used for measuring anxiety. Propensity score matching was used to adjust for differences in baseline characteristics among the four surveys. Wilcoxon signed ranks test was used to compare people's perceived risk, anxiety and protective behaviors changes in four stages. General linear model was used to identify associations between some demographic factors and perceived risk, anxiety scores, and protective behaviors.

**Results:** The proportion of high perceived risk has dropped from 24.7 to 4.7%. The proportion of severe anxiety has declined from 12.2 to 1.2%. The proportion of people wore masks when they went out has increased from 97.0 to 98.3%. Women were more likely to develop anxiety (OR = 1.5, 95%CI: 1.4–1.6) and more positively adopted recommended behaviors (OR = 2.1, 95%CI: 1.3–3.4) than men. People at age 30–39 years, with high-degree education, with married status, and accompanied with poor self-rated health status were prone to have higher risk perception and anxiety. Perceived risk was significantly associated with anxiety over the entire periods. Anxiety levels had stronger associations with adoption of protective behaviors (wearing mask and avoiding crowed place) in the early epidemic periods than in the late epidemic periods.

**Conclusions:** The levels of perceived risk and anxiety showed a trend of rising first and then falling. Gradually upward trend on initiative preventive behaviors including wearing mask and avoiding visiting crowded places also was observed through scanning data at four stages. People at age 30–39 years, with high-degree education, and accompanied with poor self-rated health status were prone to have higher risk perception and anxiety. Our findings showed that people simultaneously presented both high-level risk perception and anxiety across the four wave surveys, leading to their positive self-prevention and protective behavior.

## Introduction

The COVID-19 pandemic has created an unprecedented crisis (1). The first wave of the COVID-19 epidemic in China was brought under control with 3 months—from mid-January 2020 (when human-to-human transmission of COVID-19 was confirmed) to the end of March 2020 (2). COVID-19 was recognized as a Class B infectious disease by National Health Commission, and was treated as a Class A infectious disease for prevention and control on January 20, 2020. The World Health Organization (WHO) declared it a Public Health Emergency of International Concern on January 30, 2020 (3). Based on the number of confirmed COVID-19 cases in China, we roughly divided the epidemic into four periods: the early outbreak period, the rising period, the falling period and the controlled period. As of January 28, 2020 (when the first-round survey started), COVID-19 infection caused 5,974 cases in Mainland China. By February 10, 2020, the epidemic dramatically expanded, 40,171 cases have been reported (when the second-round survey started). When the third-round survey was conducted on February 20, 2020, 54,965 cases were reported. And 80,026 cases have been reported in Mainland China as of March 1, 2020 (when the fourth-round survey started). At that time in our fourth-round survey, the epidemic was relatively under control, and there was a downward trend in the number of new cases per day. By end of October 2021, more than 273 million confirmed COVID-19 cases were detected in 216 countries, territories, and areas and more than 4.84 million deaths have been reported (4) (see Supplementary Appendix 1 for detailed timeline).

The COVID-19 pandemic has yielded a series of undesirable effects on all aspects of society, including physical health and mental health (5, 6). According to stage theory, risk perception acts as a trigger for precautionary action (7, 8). Previous studies suggested that people with higher risk perceptions were more likely to take comprehensive precautionary measures against infection (9, 10). The China National Health Commission released eight versions of the new coronavirus pneumonia prevention and control protocol (11), and published guidelines for public prevention of coronavirus including minimizing outings, wearing masks, keeping hands clean, and avoiding crowded places (12). Accordingly, risk perception also affects public psychology states (13). During an outbreak of an infectious disease, individuals often change their behavior to reduce their risk of infection. Previous studies found that risk perceptions of infection can be predictors of a range of preventive behaviors during an emergency pandemic (14, 15). The levels of risk perception of infection greatly influence emotional concern involving anxiety and subsequent preventive behaviors. Recognizing the significance of these differences may be beneficial in developing practical interventions when attempting to motivate particular groups to practice preventive measures during outbreaks. Although risk perception of infection can be a predictor of preventive behaviors, excessive risk perception can increase the likelihood of negative affective (e.g., anxiety and panic) occurring (16, 17). Emotional anxiety can potentially contribute to “emotional contagion” between groups during a time of collective concern. It is important to understand the individual factors that predict anxiety to avoid the occurrence of clinically significant anxiety. Moreover, there exists a strong correlation between family and friends' response to outbreak and personal preventive behaviors, underlining the herd behavior of individuals (18). Therefore, there is a particular need to examine the differences of risk perception, emotional anxiety, and behavior response over time during the pandemic.

Although previous studies revealed that anxiety was strongly associated with demographic factors and the perceived risk (19, 20), these studies consisted of single, cross-sectional surveys, and did not account for the time scale effects during COVID-19 pandemic. In addition, the COVID-19 pandemic brings a new challenge to public emergency management, demanding consideration of not only the traditional cognitive estimates of risk but of the significant role emotional anxiety plays in predicting behavioral outcomes with the time scale effects (21–23).

In this study we aimed to examine the risk perception, anxiety, and behavioral response related to the COVID-19 pandemic in the general Chinese population. The aims of this study were (1) to identify trends over time in perceived risk, anxiety, and behavior response and (2) to assess factors significantly associated with perceived risk, anxiety, and behavioral response (e.g., preventive measures such as wearing mask and avoiding visiting crowded places).

## Materials and Methods

### Study Population

**Figure 1:**
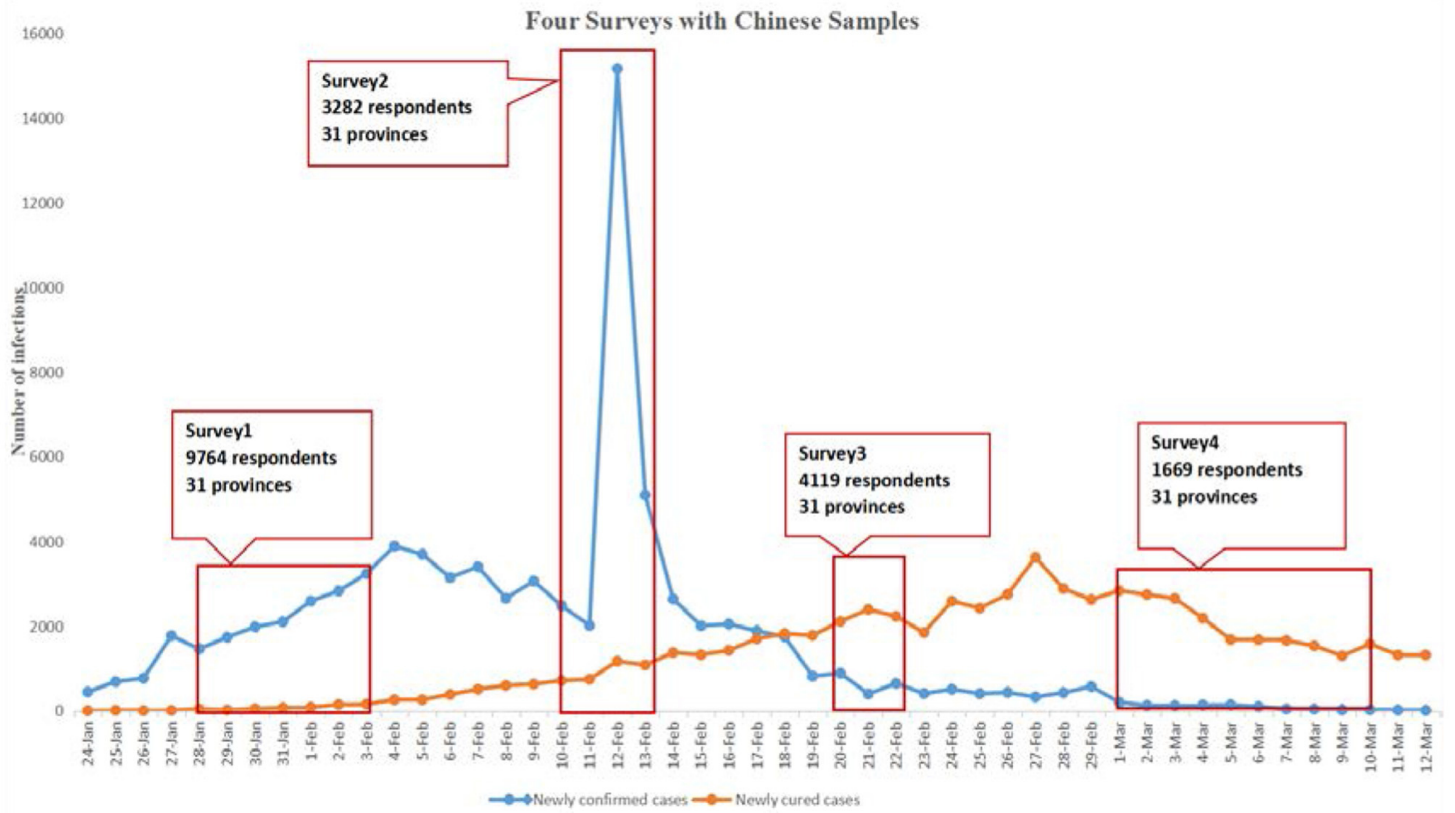
The study overview.

Four cross-sectional, population-based, online surveys were conducted, the first survey (S1) was from January 28 to February 3, 2020, the second survey (S2) was from February 10 to 12, 2020, the third survey (S3) was from February 20 to 22, 2020, and the fourth survey (S4) was from March 1 to 10, 2020. They were open online questionnaires for the people (1) aged ≥18 years, (2) resides in China, (3) willing to respond, (4) able to complete the questionnaire by mobile phone or computer. We use PASS (Power Analysis and Sample Size, Version: 15.0.5, NCSS Statistical Software, United States) to calculate the necessary sample size on the basis of an expected minimal change of 5% in people's attention to the epidemic, psychological effect, and individual prevention practice with α: 0.05 and β: 0.20. In this study, 1,047 participants at most were required. Considering a possible dropout rate of 20%, at least 1,309 participants in total (see Supplementary Appendix 2). The study overview is summarized in Figure 1.

### Online Questionnaire

We designed a structured Chinese questionnaire and collected data on Wenjuanxing, an online platform that provides functions equivalent to Amazon Mechanical Turk. Convenience sampling was used for easy recruiting survey participants under the long-term impact of the COVID-19 epidemic. The four independent online questionnaires were sent from the same approach (WeChat and MicroBlog), and anyone who receives the questionnaire on the Internet or mobile phone and meets the inclusion criteria could fill in it. After a large number of questionnaires were collected, some samples were excluded according to the exclusion criteria. The same questionnaires repeatedly used in the four surveys, mainly including the following information: (1) socio-demographic information of respondents; (2) frequency of attention to COVID-19; (3) practices of preventive measures against COVID-19, including wearing masks, keeping physical distance, personal hygiene practices, and keeping the indoor and living environment clean; (4) anxiety toward COVID-19; and (5) perceived risks. The questionnaire consisted of 25 questions on average and could be completed in 3–5 min.

#### Socio-Demographic Variables

Demographic information collected included age, sex, marriage, education, occupation, area, family members, and residence. Variables related to COVID-19 contact history included close contact with an individual with confirmed COVID-19, indirect contact with an individual with confirmed COVID-19 and contact with an individual with suspected COVID-19 or infected materials.

#### Frequency of Attention to COVID-19

Respondents expressed their degree of concern about the situation related to COVID-19 by one-item: “To what extent are you concerned about the current COVID-19-linked situation?” A five points Likert-type scale were used to ascertain the frequency of attention to COVID-19 (from 1 to 5, 1 = never, 2 = little, 3 = sometimes, 4 = often, and 5 = always).

#### Preventive Measures

COVID-19 preventive measures practices included wearing masks, avoiding crowded places, personal hygiene practices, and keeping the indoor and living environment clean were measured with dichotomous variables. Questions were scored “1 point” (yes) or “0 points” (no).

#### Anxiety Toward COVID-19

Participants' anxiety was measured via a five-item short form of the State Scale of the Spielberger state-trait anxiety inventory (STAIS-5) (24) and modified for Chinese Context. Participants answered on a four-point scale (0–3 points) for each item. The total anxiety score was divided into normal (0–6), mild anxiety (7–9), moderate anxiety (10–13), severe anxiety (14, 15). Someone scoring ≥14 on the STAIS-5 was considered potentially clinically anxious.

#### Risk Perception

Perceived risk was assessed based on previous studies conducted among the general population (25), with one item: “How likely do you think it is that you will get COVID-19?” Risks were divided into five categories: 1 = no risk, 2 = low risk, 3 = medium risk, 4 = high risk, 5 = extremely high risk.

#### Subjective Health Status

Subjective health status was measured via one item: “How would you define your health status?” Health status was divided into four categories: 1 = unhealthy, 2 = ordinary, 3 = good healthy, 4 = very healthy.

### Data Management and Statistical Analysis

We used SPSS (version 20.0, IBM Corp, Armonk, NY) and STATA (version 15.1, Stata Corp LLC, College Station, Texas, USA) for data cleaning and statistical analysis. Categorical variables were expressed as absolute and relative frequencies. Perceived risk scores, anxiety scores, and measure practices scores were age-standardized using the China population in 2019 and the direct standardization method. Propensity score matching was used to adjust for differences in baseline characteristics among the four surveys. Matching was performed with the use of a 1:1 matching protocol without replacement (greedy–matching algorithm), with a caliper width equal to 0.2 of the standard deviation of the logit of the propensity score. In the matched cohort, paired comparisons were performed with the use of McNemar's test for binary variables. Wilcoxon signed ranks test was used to compare perceived risk score, anxiety score, and preventive measures score changes in four stages. General linear model was used to analyze associations between socio-demographic factors and perceived risk, anxiety scores, and preventive measures. Odds ratio (OR) and their 95% confidence intervals (CI) were calculated as estimates of the correlations. All *p*-values were two-sided and *p* < 0.05 was considered statistically significant.

### Quality Control

We monitored the progress of the survey daily. After the deadline, we checked the accuracy of data, and excluded the questionnaires if (1) the age range was below 18; (2) the answering time was <150 s; or if there were (3) logical contradictions between the answers to the questionnaire. All data were checked for consistency by two research members.

### Ethical Approval

This study was approved as ethically exempt by the Peking University Health Science Center Ethics Committee (IRB00001052). All subjects participated in the surveys voluntarily, and the information in the database was completely de-identified.

## Results

### Study Participants and Characteristics

Eleven thousand one hundred thirty-eight individuals participated in S1. Among these, 1,374 were excluded due to out of age range or incomplete questionnaire, and the effective rate was 87.7% (9,764/11,138). Three thousand five hundred ninety-seven individuals participated in S2. Among these, 315 were excluded due to out of age range or incomplete questionnaire, and the effective rate was 91.2% (3,282/3,597). Four thousand four hundred and fifty individuals participated in S3. Among these, 331 were excluded due to answering without serious consideration or out of age range or incomplete questionnaire, and the effective rate was 92.6% (4,119/4,450). One thousand nine hundred thirty-eight individuals in S4. Among these, 269 were excluded due to answering without consideration or out of age range or incomplete questionnaire, and the effective rate was 86.1% (1,669/1,938). The total effective rate was 89.2% (18,834/21,123).

The participants covered 30 provincial administrative regions in Mainland China. Six thousand four hundred and ninety-one (34.5%) respondents were male; 5,648 (30.0%) respondents were younger than 30 years old; 15,459 (82.2%) respondents were with bachelor's degree or above; 5,617 (29.8%) were unmarried; and 2,223 (11.8%) were from Wuhan city (Table 1).

**Table 1:** Baseline characteristics of participants in four online surveys.

	**Survey 1**		**Survey 2**		**Survey 3**		**Survey 4**		**Total**	
	***N***	**%**	***N***	**%**	***N***	**%**	***N***	**%**	***N***	**%**
**Gender**
Male	3,271	33.5	1,017	31.0	1,435	35.0	768	46.0	6,491	34.5
Female	6,493	66.5	2,265	69.0	2,668	65.0	901	54.0	12,327	65.5
**Age**
<30 years	3,124	32.0	899	27.4	1,241	30.2	384	23.0	5,648	30.0
30–39 years	2,803	28.7	1,084	33.0	1,079	26.3	472	28.3	5,438	28.9
40–49 years	2,236	22.9	876	26.7	1,070	26.1	437	26.2	4,619	24.5
≥50 years	1,611	16.5	423	12.9	713	17.4	376	22.6	3,123	16.6
**Education**
Junior high school and below	449	4.6	169	5.1	166	4.0	87	5.2	871	4.6
Senior high school	1,269	13.0	485	14.8	550	13.4	174	10.4	2,478	13.2
Bachelor's degree	5,575	57.1	2,122	64.7	2,586	63.0	986	59.1	11,269	59.9
Master's degree or above	2,461	25.2	506	15.4	801	19.5	422	25.2	4,190	22.3
**Marriage**
Unmarried	3,154	32.3	895	27.3	1,179	28.7	389	23.3	5,617	29.8
Married	6,278	64.2	2,215	67.5	2,734	66.6	1,228	73.6	12,455	66.2
Divorced	244	2.5	143	4.4	165	4.0	38	2.3	590	3.1
Other	88	0.9	29	0.9	25	0.6	14	0.8	156	0.8
**Occupation**
Medical professional	1,758	18.0	457	13.9	1,099	26.8	249	14.9	3,563	18.9
Labors	674	6.9	29	0.9	52	1.3	150	9.0	905	4.8
Teachers and researchers	1,843	18.9	339	10.3	398	9.7	416	24.9	2,996	15.9
Government staff	391	4.0	312	9.5	526	12.8	85	5.1	1,314	7.0
C&S personnel	1,934	19.9	778	23.7	814	19.8	312	18.7	3,838	20.4
Students	1,533	15.7	373	11.4	669	16.3	162	9.7	2,737	14.5
Other^#^	1,631	16.7	994	30.3	545	13.3	295	17.7	3,465	18.4
**Residence**
Urban	7,811	80.0	2,564	78.1	3,151	76.8	911	54.6	14,437	76.7
Rural	1,953	20.0	718	21.9	952	26.2	758	45.4	4,381	23.3
**Area**
Wuhan city	101	1.0	634	19.3	1,461	35.6	27	1.6	2,223	11.8
Hubei province	55	0.6	282	8.6	693	17.0	12	0.7	1,042	5.5
Other provinces in China	9,408	96.4	2,340	71.3	1,933	47.1	1,630	97.7	15,311	81.4
Abroad	200	2.0	26	0.8	16	0.4	0	0.0	242	1.3
**Health Status**
Very healthy	5,683	58.2	1,302	39.7	2,171	52.9	564	33.8	9,720	51.7
Good healthy	3,105	31.8	1,458	44.4	1,615	39.4	880	52.7	7,058	37.5
Ordinary	751	7.7	491	15.0	291	7.1	185	11.1	1,718	9.1
Unhealthy	225	2.3	31	1.0	26	0.6	40	2.4	322	1.7
**Total**	9,764	100.0	3,282	100.0	4,103	100.0	1,669	100.0	18,818	100.0

*C&S, Commercial and service personnel*.

# *Including farmer, civil servant, self-employed, driver, retired people, unemployed, etc*.

### Time-Trends in Perceived Risk, Anxiety and Measures Taken

In S1, 97.0% of people (3,238/9,764) paid daily attention to the epidemic. But in S4, the proportion of people who paid daily attention to it had dropped to 88.5% (1,477/1,669). The change was statistically significant (*P* < 0.0001). The proportion of high perceived risk has decreased significantly from 24.7% inS2 to 4.7% in S3 (*P* < 0.0001). Similarly, the proportion of severe anxiety has declined from 12.2% in S2 to 1.2% in S3 (*P* < 0.0001). The proportion of people wore masks when they went out and hand hygiene has increased from S1 to S4 (from 97.0 to 98.3% for wearing mask, from 91.3 to 96% for hand hygiene), and the increase is statistically significant (*P* = 0.0034 for wearing mask, *P* < 0.001 for hand hygiene). The proportion of avoiding crowded places has declined from 98.5% in S1 to 94.4% in S4 (*P* < 0.0001) (Table 2).

**Table 2:** Trends over time in perceived risk, anxiety and preventive measures.

	**Survey1**	**Survey2**	**Survey3**	**Survey4**	**Time trend**
	**(28 January−3 February)**	**(10 February−12 February)**	**(20 February−22 February)**	**(1 March−10 March)**	**1 vs. 4**	***P***	**2 vs. 3**	***P***
**Frequency of attention to the epidemic (%)**	3,238 (97.0)	1,229 (84.5)	1,120 (77.0)	1,477 (88.5)	–	<0.0001	–	<0.0001
**Perceived risk (%)**					–	<0.0001	–	<0.0001
No risk	677 (20.3)	314 (21.6)	607 (41.7)	582 (34.9)				
Low risk	1,993 (59.7)	201 (13.8)	446 (30.7)	877 (52.5)				
Medium risk	471 (14.1)	343 (23.6)	317 (21.8)	159 (9.5)				
High risk	147 (4.4)	360 (24.7)	69 (4.7)	48 (2.9)				
Extremely high risk	50 (1.5)	237 (16.3)	16 (1.1)	3 (0.2)				
**Anxiety (%)**					–	<0.0001	–	<0.0001
Normal	1,476 (44.2)	219 (15.1)	602 (41.4)	1,103 (66.1)				
Mild anxiety	1,088 (32.6)	180 (12.4)	291 (20.0)	274 (16.4)				
Moderate anxiety	700 (21.0)	878 (60.3)	544 (37.4)	252 (15.1)				
Severe anxiety	74 (2.2)	178 (12.2)	18 (1.2)	40 (2.4)				
**Measures taken (Do it, %)**								
Wearing mask	3,238 (97.0)	1,404 (96.5)	1,412 (97.0)	1,641 (98.3)	+	0.0034	+	0.463
Avoiding crowded places	3,288 (98.5)	1,415 (97.3)	1,395 (95.9)	1,576 (94.4)	–	<0.0001	–	0.0532
Hand hygiene	3,048 (91.3)	1,377 (94.6)	1,368 (94.0)	1,602 (96.0)	+	<0.0001	–	0.524
Keeping the indoor and living environment clean	2,443 (73.2)	1,132 (77.8)	1,304 (89.6)	1,132 (67.8)	–	<0.0001	+	<0.0001

### Region Difference in Perceived Risk, Anxiety, and Preventive Measures

In east region, with the change of time and epidemic, the level of perceived risk showed a first rising and then decline trend (*P* < 0.001). Similarly, the level of anxiety from S1 to S2 mainly revealed an upward trend, and the level of the anxiety showed a downward trend from S2 to S3 (*P* < 0.001). The preventive measures score showed a trend of being relatively stable and then slightly increased (*P* < 0.001).

In central region, with the change of time and epidemic, the level of perceived risk showed a first rising and then decline trend (*P* < 0.001). Similarly, the level of anxiety from S1 to S2 mainly revealed an upward trend, and the level of the anxiety showed a downward trend from S2 to S3 (*P* < 0.001). The preventive measures score showed a trend of being relatively stable and then slightly increased (*P* < 0.001).

In west region, with the change of time and epidemic, the level of perceived risk showed a first rising and then decline trend (*P* < 0.001). Similarly, the level of anxiety from S1 to S2 mainly revealed an upward trend, and the level of the anxiety showed a downward trend from S2 to S3 (*P* < 0.001). The preventive measures score showed a trend of being relatively stable and then slightly increased (*P* < 0.001) (Table 3).

**Table 3:** Region difference in perceived risk, anxiety and preventive measures.

	**Survey 1**		**Survey 2**		**Survey 3**		**Survey 4**		***P*-values**
	***N***	**Median (P_**25**_, P_**75**_)**	***N***	**Median (P_**25**_, P_**75**_)**	***N***	**Median (P_**25**_, P_**75**_)**	***N***	**Median (P_**25**_, P_**75**_)**	
**Perceived risk score**									
East region	5,146	2.0 (2.0–2.0)	634	3.0 (2.0–4.0)	970	2.0 (1.0–3.0)	875	2.0 (1.0–2.0)	<0.001
Central region	1,533	2.0 (2.0–3.0)	2,366	3.0 (3.0–4.0)	2,669	2.0 (1.0–3.0)	330	2.0 (1.0–2.0)	<0.001
West region	3,085	2.0 (2.0–3.0)	282	3.0 (1.0–4.0)	464	2.0 (1.0–2.0)	464	1.0 (1.0–2.0)	<0.001
**Anxiety score**									
East region	5,146	7.0 (3.0–10.0)	634	12.0 (10.0–13.0)	970	8.0 (5.0–10.0)	875	7.0 (4.0–9.0)	<0.001
Central region	1,533	7.0 (3.0–10.0)	2,366	12.0 (10.0–13.0)	2,669	8.0 (5.0–10.0)	330	8.0 (5.0–10.0)	<0.001
West region	3,085	6.0 (3.0–10.0)	282	11.0 (10.0–13.0)	464	7.0 (4.0–10.0)	464	7.0 (4.0–9.0)	<0.001
**Preventive measures score**									
East region	5,146	4.0 (3.0–4.0)	634	4.0 (3.0–4.0)	970	4.0 (3.0–4.0)	875	4.0 (4.0–4.0)	<0.001
Central region	1,533	4.0 (3.0–4.0)	2,366	4.0 (3.0–4.0)	2,669	4.0 (3.0–4.0)	330	4.0 (4.0–4.0)	<0.001
West region	3,085	4.0 (3.0–4.0)	282	4.0 (3.0–4.0)	464	4.0 (3.0–4.0)	464	4.0 (4.0–4.0)	<0.001

### Factors Associated With Perceived Risk, Anxiety, and Preventive Measures

Generalized linear models were performed to identify factors significantly associated with (1) perceived risk, (2) anxiety, (3) behavior in wearing mask, and (4) avoiding visiting crowded places. In this regression analysis variables of the survey in four periods (S1, S2, S3, and S4) were included.

**Figure 2:**
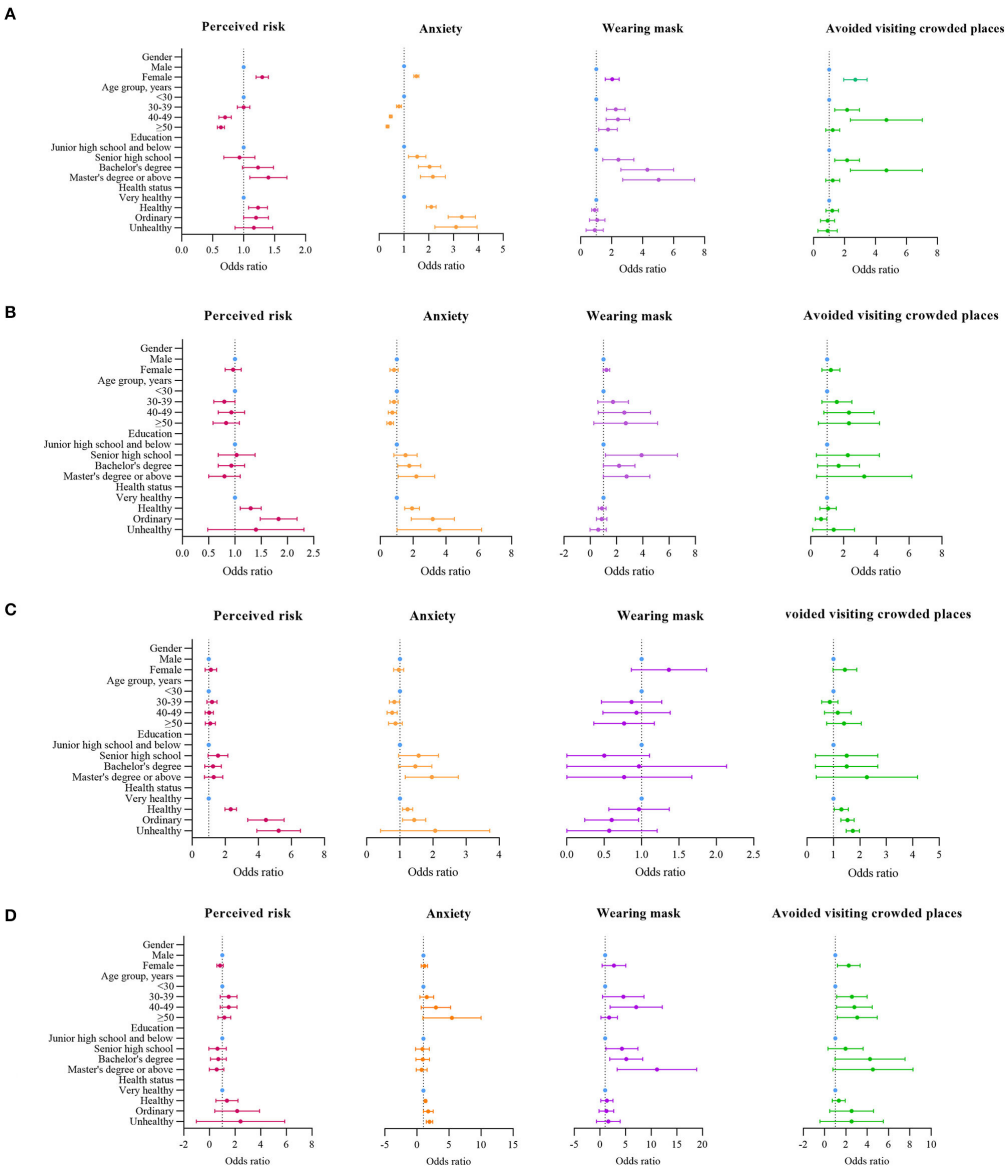
Factors associated with perceived risk, anxiety, and preventive measures. **(A)** Odds ratios comparing with different characteristics the rate of perceived risk, anxiety, and preventive measures on S1. **(B)** Odds ratios comparing with different characteristics the rate of perceived risk, anxiety, and preventive measures on S2. **(C)** Odds ratios comparing with different characteristics the rate of perceived risk, anxiety, and preventive measures on S3. **(D)** Odds ratios comparing with different characteristics the rate of perceived risk, anxiety, and preventive measures on S4.

The general linear model illustrated that women had a higher perceived risk than men for S1 (OR = 1.3, 95% CI: 1.2–1.4) (Figure 2A). Compared to those <30 years, those aged 40–49 years and those >50 years had a lower perceived risk (OR = 0.6, 95% CI: 0.6–0.7). In S3 (Figure 2C), compared to very healthy people, people who were unhealthy had a higher perceived risk about the outbreak (OR = 8.5, 95% CI 3.8–19.4).

Over the entire period, women, younger people, those with a bachelor's degree and above, and those with poor health status were more likely to experience anxiety. Women were more likely to experience anxiety compared to men (OR = 1.5, 95% CI: 1.4–1.6) (S1, Figure 2A). The most anxiety prone group was younger people under 30 years of age. In S4, those over 50 years of age were more likely to experience anxiety compared to those under 30 years of age (OR = 4.2, 95% CI: 1.7–10.5). In S2, compared to people with a junior high school and below education, people with a bachelor's degree or ≥master's degree were more likely to be anxious about the outbreak (OR = 1.7, 95% CI: 1.1–2.5; OR = 2.0, 95% CI: 1.2–3.4, respectively) (Figure 2B). In S3, people with poor health status were more likely to experience anxiety (OR = 3.0, 95% CI: 2.3–4.0) (Figure 2C).

In S1, compared to men, women were more likely to wear masks (OR = 2.0, 95% CI: 1.6–2.5) (Figure 2A) and avoid crowded places (OR = 2.1, 95% CI: 1.3–3.4) (Figure 2D). People ≥30 years of age were more likely to wear masks compared to those under 30 years of age (OR = 2.2, 95% CI: 1.7–2.9 for those aged 30–39 years; OR = 2.3, 95% CI: 1.7–3.2 for those aged 40–49 years) (Figure 2A). In S4, people ≥30 years of age were more likely to avoid crowded places compared to those under 30 years of age (OR = 2.3, 95% CI: 1.3–4.1 for those aged 30–39 years; OR = 2.5, 95% CI: 1.3–4.6 for those aged 40–49 years; and OR = 2.7, 95% CI: 1.4–5.1 for those aged ≥50 years) (Figure 2D). In S1, compared to people with a ≤ junior high school education, people with a bachelor's degree or ≥master's degree were more likely to wear mask (OR = 4.1, 95% CI: 2.7–6.1 for bachelor's degree; OR = 4.7, 95% CI: 2.9–7.5 for master's degree or above) (Figure 2A). In S4, compared to people with a ≤ junior high school education, people with a bachelor's degree or ≥master's degree were more likely to avoid crowded places (OR = 3.4, 95% CI: 1.5–7.9 for bachelor's degree; OR = 3.5, 95% CI: 1.4–8.7 for master's degree or above) (Figure 2D).

### Anxiety-Behavioral Associations Across Different COVID-19 Epidemic Periods

**Figure 3:**
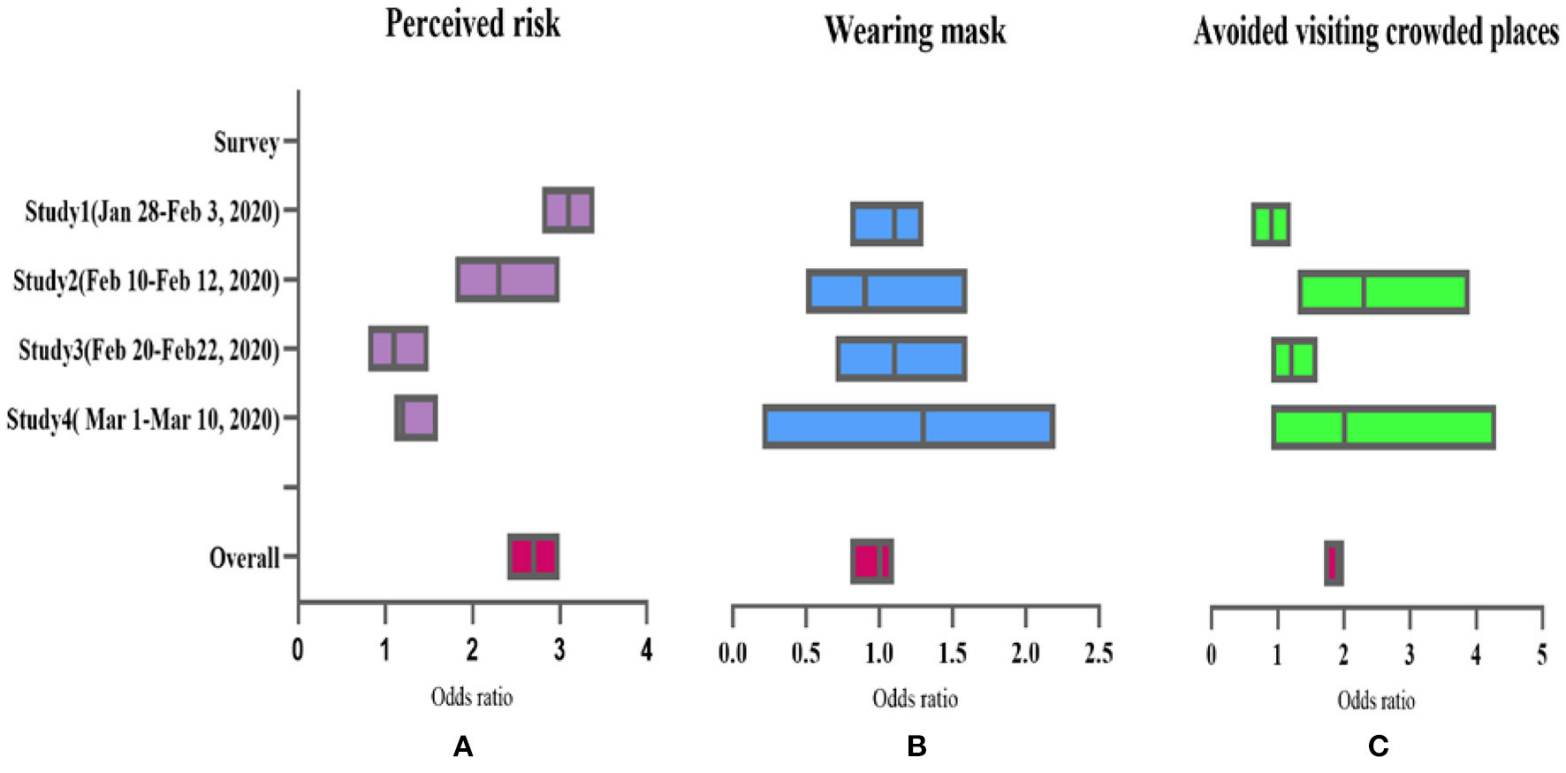
Anxiety-behavioral associations across different COVID-19 epidemic periods. **(A)** The association of perceived risk and anxiety in S1, S2, S3, and S4. **(B)** The association of wearing mask and anxiety in S1, S2, S3, and S4. **(C)** The association of avoided visiting crowded places and anxiety in S1, S2, S3, and S4.

Figure 3 shows forest plots describing the association between state anxiety with risk perception, wearing mask, or avoiding visiting crowded places for the four surveys. Figure 3A shows that state anxiety was significantly associated with perceived risk in S1, S2, S3, and S4. The association is consistently positive and statistically significant across the four surveys. After adjusting for age, sex, education, the overall OR is 2.0 (95% CI: 1.82–2.27) (*P* < 0.001). Figure 3B shows that state anxiety was not significantly associated with wearing mask in S1, S2, S3, and S4. After adjustment for age, sex, education, the overall OR is 1.0 (95% CI: 0.8–1.1.1). Figure 3C shows that state anxiety was significantly associated with avoiding visiting crowded places in S1 and S3. After adjustment for age, sex, education, the overall OR is 1.8 (95% CI: 1.6–1.9) (*P* < 0.01).

## Discussion

The COVID-19 pandemic presented health, economic, and social lost (26). We performed four cross-sectional surveys during the pandemic. The studies covered the entire phase of the COVID-19 pandemic in China. At the early outbreak phase, we found that participants experienced varying degrees of anxiety. The degree of anxiety was reduced as the outbreak became effectively controlled. The result was consistent with those reported during the SARS and H1N1 outbreak (19, 27). The risk perception significantly decreased from the early outbreak period to the under controlled period; however, people were likely to wear mask and avoid visiting crowded places. While previous studies were mainly conducted in early pandemic periods (28, 29), this study examined affective-behavioral associations across the entire wave of the COVID-19 outbreak in China and found that the association between anxiety and adoption of protective behaviors were consistently strong and positive across the different pandemic periods in China.

This study found a decreasing trend over four study periods in the level of perceived risk and presence of anxiety and an increasing trend in preventive behaviors including wearing mask and avoiding visiting crowded places. Moreover, there is no difference among the East, Central, and West in China in these measures. The findings indicate that the level of perceived risk and presence of anxiety were gradually alleviated over time. These trends may be correlated with increased cognitive awareness toward the COVID-19 pandemic or with China's effective prevention and control measures and high-level trust between the public, government and scientists. As Figure 1 and Table 2 shows, the variation in trends of the outcomes were correlated with time, but not with newly confirmed cases.

This study demonstrated that in the early stage of the outbreak, women had a higher level of risk perception than men as supported by the literatures (30, 31). One possible reason is that women are more sensitive to the risk of COVID-19 and more easily influenced by the environment. Our findings showed that women were more likely to experience anxiety than men, which was consistent with a survey in England and in Spain in March 2020. The reason may be that there were a greater number of sources of pressure for women compared to men such as having to do unpaid work caring for children and dependent relatives. Women are more emotionally vulnerable to the effects of COVID-19 than men (32). Another finding of this study showed that females preferred to take positive preventive measures including wearing mask and avoiding visiting crowded places. Similar finding on Qatari general population was also reported during the COVID-19 pandemic (33). The findings suggested there were gender differences in the precautionary behaviors to avoid contagion, indicating that women more likely than men to adopt recommended behaviors and were more likely to practice social distancing and adopt protective behaviors.

Considering age, the results showed that people younger than 30 years were more likely to have higher levels of risk perception compared to the other age groups in the early stage and peak of the epidemic. A similar study supported our findings and found that one in three U.S. young adults reported clinical cut-off symptoms of panic, anxiety as well as depression (34). As a worldwide stressor, the COVID-19 pandemic created an uncertain environment in that there was not a foreseeable endpoint to the pandemic, and relative followed effects included various domains (e.g., financial, relational, and health). Young adults were more likely to understand these effects, which contributed to higher mental issues, due to higher overall exposure to information through multiple information channels including social media and office media (35). Moreover, first-time and inaccurate information aggravated an already-fragile psychological balance of younger adults (36), causing immense fear and uncertainty, and in turn leading to greater risk perception and anxiety. As young adults tend to timely obtain more information and resource through online channels, demonstrate higher perception than other. At same time, they prefer to spread the messages to other family member to prevent their family from the infection of COVID-19. One Romania study found that many young parents prohibited their children to carry out educational and recreational activities because of excessive concern for COVID-19 infection (37). Another Egypt study found that healthcare workers had lacked confidence to protect themselves and their families during the COVID-19 pandemic due to wider social networks and professional information resources (38). These findings is consistent with the findings of previous studies (39–41), but contrary to a Portuguese study that reported older individuals have a higher risk perception for mental disorders in the state of emergency (42). Meanwhile, we found that persons of greater age were more likely to have positive preventive behavior such as wearing masks and avoiding visiting crowded places. In China, central and local governments have been implementing strict regulations that people must wear facial masks in a public space (43). Meanwhile, Chinese people were prone to consciously practice precautionary behaviors, especially the middle aged and the elderly groups who were warned that they were at the greatest risk of COVID-19 related mortality. At the controlled stage of the COVID-19 outbreak, the Chinese government still encourage people to take enhanced personal protective measures, including wearing mask and avoiding crowded places.

Among factors influencing risk perception, psychological response, and preventive measures, people with higher levels of education were more likely to have higher level of risk perception and experience anxiety. Higher-level educated people were more likely to adopt preventive measures like wearing masks or avoiding crowded places. A study from Saudi Arabia reported similar results; higher-level educated participants were more likely to adopt protective practices (44). The results could be interpreted by assuming that risk perception as well as risk communication about disease severity appear to increase the chances of successfully implementing protective measures. However, the overwhelming amount of negative information and the overuse of mass media in communicating the pandemic might contribute to “media storms” and “infodemics” in response to COVID-19 (45), leading to overreaction, unwarranted public fear, and an overly pessimistic feeling in the perceived current risk (46).

Our findings showed that people who had a poor self-rated health status had higher perceived risk about the outbreak as well as higher anxiety levels; a good self-rated health status was not associated with practicing protective behaviors. Similar findings were seen in other studies (46, 47). As vulnerable populations in the current pandemic, the general consensus was that patients with multiple comorbidities are at higher risk of COVID-19 mortality than those healthy people (48, 49). Therefore, if they confirmed themselves to have comorbidities, they also are more likely to consider themselves to have a high risk for COVID-19 infection and more vulnerable to the development of mental disease, such as anxiety and depression (50). This finding suggests that health authorities should pay particular attention to those with poor health status and should provide enough resources for psychological support and interventions. The results of this study can be used to guide the development of strategies targeting preventive measures in vulnerable populations with a higher risk of mental health.

Our findings showed that people simultaneously presented with both high-level risk perception and anxiety across the four surveys, leading to positive prevention behavior of avoiding visiting crowded places. With a consistent finding, a similar study found that higher perceived COVID-19 risk predicted greater mental problem (51). A significant negative correlation between preventive behaviors and risk perception was also shown in Iranian study (52). As a foremost recommended prevention measure, avoiding visiting crowded places is a key part of decreasing the spread of COVID-19 (53). The anxious people prefer to wear masks to protect others and themselves (54). The findings of this study have significant public health implications in that they strengthen the classification of psychological and behavior interventions for an effective response to the pandemic.

## Limitations

Some limitations in the current study must be acknowledged. First, a convenience sample was adopted to collect data, which increased the potential for sampling bias. Second, the cross-sectional study cannot effectively and precisely judge the causal relationship between the variables. Moreover, four cross-sectional surveys were not equivalent to a longitudinal study. Third, the data were collected from self-reports from participants by means of an online survey, which is likely to introduce information bias from social desirability or negative affection. Fourth, several single-item tools in this study were used for collecting the data in order to abbreviate survey material and potentially increase response rates, but this may have reduced the validity and reliability of the measurements; therefore, a widely used measurement tool should be adopted in the future.

## Conclusions

The levels of perceived risk and anxiety showed a trend of rising first and then falling, indicating that psychological and mental issues caused by the COVID-19 pandemic gradually subsided over time. Additionally, the proportion of people practicing preventive behaviors such as wearing a mask and avoiding visiting crowded places also increased over the four survey periods, indicating that these behaviors gradually became a conscious habit. Women were more likely to experience anxiety, adopt recommended preventive behaviors, and practice social distancing than men. People aged <30 years, with high-degree education, or with poor self-rated health status were more likely to have higher risk perception and more likely to experience anxiety. Additionally, they also were more likely to practice positive preventive behaviors. Our findings showed that people simultaneously presented both high-level risk perception and anxiety across the four surveys, leading to their positive self-prevention and protective behavior. The findings contribute to the suggestion that health authorities and policy-makers should pay particular attention to those who are vulnerable and provide support and interventions related to psychological and mental health. The results in the current study can be used to guide the development of preventive strategies in vulnerable populations with a higher perceived risk of psychological and mental health.

## Data Availability Statement

The original contributions presented in the study are included in the article/Supplementary Material, further inquiries can be directed to the corresponding author/s.

## Ethics Statement

This study was approved as ethically exempt by the Peking University Health Science Center Ethics Committee (IRB00001052). All subjects participated in the surveys voluntarily, and the information in the database was completely de-identified.

## Author Contributions

BL and FC: conceived and designed the study and approved the final manuscript for publication. BL, HL, and TS: performed the study. BL, BH, HL, and TZ: analyzed the data. BL, BH, HL, and XT: contributed reagents, materials, and analysis tools. BL, TS, and FC: wrote the paper. All authors have read and approved the manuscript.

## Funding

This work was supports by Fundamental Research Funds for the Central Universities and Peking University Health Science Center (grant numbers BMU20170607).

## Conflict of Interest

The authors declare that the research was conducted in the absence of any commercial or financial relationships that could be construed as a potential conflict of interest.

## Publisher's Note

All claims expressed in this article are solely those of the authors and do not necessarily represent those of their affiliated organizations, or those of the publisher, the editors and the reviewers. Any product that may be evaluated in this article, or claim that may be made by its manufacturer, is not guaranteed or endorsed by the publisher.
