# Supplementary figures and images for: Trends and Factors Associated With Risk Perception, Anxiety, and Behavior From the Early Outbreak Period to the Controlled Period of COVID-19 Epidemic: Four Cross-Sectional Online Surveys in China in 2020
Source: Front Public Health. 2022 Jan 18;9:768867. doi: 10.3389/fpubh.2021.768867 (PMC8805284; doi:10.3389/fpubh.2021.768867)

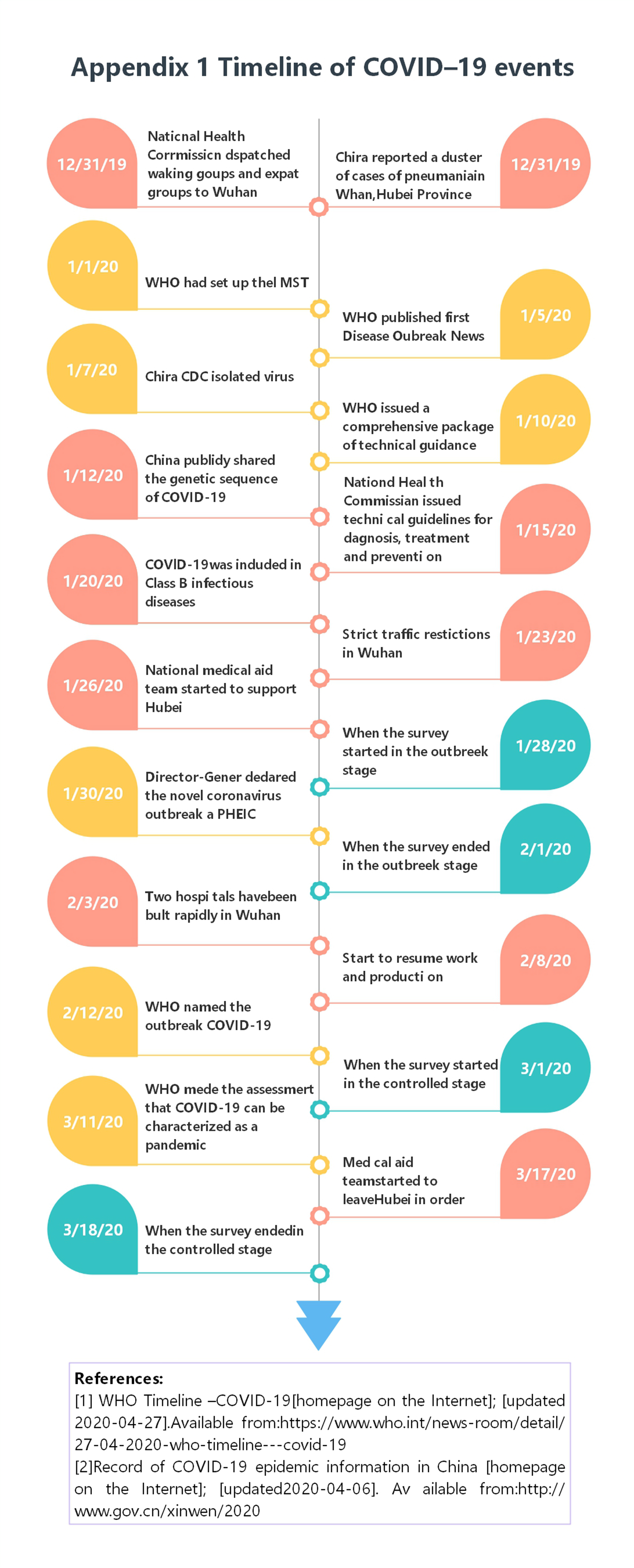

Supplement: Supplementary file 1 [file Image_1.jpg]
