# Supplementary material for: Trends and Factors Associated With Risk Perception, Anxiety, and Behavior From the Early Outbreak Period to the Controlled Period of COVID-19 Epidemic: Four Cross-Sectional Online Surveys in China in 2020
Source: Front Public Health. 2022 Jan 18;9:768867. doi: 10.3389/fpubh.2021.768867 (PMC8805284; doi:10.3389/fpubh.2021.768867)
Supplement: Supplementary file 3 [file Data_Sheet_2.docx]

**Appendix 3**

**Questionnaire on prevention practice and psychological effect of COVID-19 in 2020**

**Informed Consent**

*There is an online survey, designed by School of public health, Peking University, aiming to understand the psychological status of Corona Virus Disease 2019(COVID-19). This survey does not involve any privacy or sensitive issues, and the results of the it will be helpful to China's epidemic management.*

**Part A**

A1. Are you currently living in an urban or rural area?

a. Urban

b. Rural

A2. What is your age?

a. [number]

A3. What was your sex at birth?

a. Male

b. Female

A4. What is your marital status?

a. Never married

b. Engaged or married

c. Separated or divorced

d. Widowed

e. Other

A5. What is your highest level of education?

a. Primary school

b. Junior high school

c. Senior school

d. Some college or a bachelors degree

e. Masters, PhD, or above

A6. What is your main occupation?

a. Labor worker (blue collar)

b. Farmer

c. Teachers and researchers

d. Service/retail

e. Civil servant

f. Health care workers

g. Individual operator

h. Office worker (white collar)

i. Driver

j. Retired people

k. Student

l. Unemployed

m. Other

A7. How many people are there in your family?

a. 1

b. 2

c. 3

d. 4

e. 5

f. 6

g. 7

h. 8

i. 9

j. 10

**Part B**

B1. How often are you concerned about the outbreak?

a. Every day

b. Occasionally

c. Don't care

B2. Where do you get information about the outbreak?

[You can choose more than one]

a. Government and professional organizations (including official websites,Weibo and WeChat)

b. News media

c. Search engines (Baidu, Google, etc.)

d. Personal social media

e. TV

f. Radio

g. Community brochure/brochure/column, etc

h. Other mass media (newspapers/magazines)

i. Other (please indicate)

B3. Were you exposed to the following situation recently?

[You can choose more than one]

a. Close contact with an individual with confirmed COVID-19

b. Indirect contact with an individual with confirmed COVID-19

c. Contact with an individual with suspected COVID-19 or infected

d. No above

B4. How confident are you combating the COVID-19 pandemic?

1. Very unconfident
2. Unconfident
3. Medium confident
4. Confident
5. Very confident

B5. How likely do you think it is that you will get COVID-19?

1. No risk
2. Low risk
3. Medium risk
4. High risk
5. Extremely high risk

B6. How would you define your health status?

1. Very healthy
2. Good health
3. Ordinary
4. Unhealthy

**Part C**

*Read each statement and then circle the option under the statement that indicates HOW YOU FEEL RIGHT NOW, that is, at this moment. There are no right or wrong answers. Do not spend too much time on any one statement but give the answer which seems to describe your present feelings best. Thank you.*

C1. How did you feel nervous about COVID-19 in the latest week?

1. Not at all

b. Somewhat

c. Moderately so

d. Very much so

C2. How did you feel upset about COVID-19 in the latest week?

1. Not at all

b. Somewhat

c. Moderately so

d. Very much so

C3. How did you feel frightened about COVID-19 in the latest week?

1. Not at all

b. Somewhat

c. Moderately so

d. Very much so

C4. How did you feel jittery about COVID-19 in the latest week?

1. Not at all

b. Somewhat

c. Moderately so

d. Very much so

C5. How did you feel confused about COVID-19 in the latest week?

1. Not at all

b. Somewhat

c. Moderately so

d. Very much so

**Part D. Individual Protection**

D1. What kind of mask do you think can effectively prevent 2019-nCoV infection?

[You can choose more than one]

a. Surgical mask

b. Medical mask (N95 and above)

c. Gauze mask

e. Activated charcoal mask

f. None of them/I don't know

D2. In which environment would you wear a mask?

[You can choose more than one]

a. Parks and roads

b. Supermarkets, malls and other crowded places

c. Small confined Spaces such as elevators

e. Hospital

f. None of them/I don't know

D3. Is there a time limit for the effectiveness of mask wearing?

a. No

b. Yes, 2-4 hours

c. 5-6 hours

d. I don't know

D4. Can hand washing prevent 2019-nCoV infection?

a. Yes

b. No

c. I don't know

D5. What measures have you taken to protect yourself from 2019-nCoV?

[You can choose more than one]

a. Wear a mask when going out

c. Do not use hands when sneezing or coughing

d. Keep hands clean by washing hands correctly and timely

e. Do not go to parties or visits, and try to avoid crowded or enclosed places

f. Eat a healthy balanced diet
